# Antitumor activity of intratracheal inhalation of temozolomide (TMZ) loaded into gold nanoparticles and/or liposomes against urethane-induced lung cancer in BALB/c mice

**DOI:** 10.1080/10717544.2016.1247924

**Published:** 2017-02-26

**Authors:** Mohamed A. Hamzawy, Amira M. Abo-youssef, Heba F. Salem, Sameh A. Mohammed

**Affiliations:** 1 Pharmacology and Toxicology Department, College of Pharmacy, Misr University for Science and Technology, 6th October City, Egypt,; 2 Pharmacology and Toxicology Department, and; 3 Pharmaceutics Department, Faculty of Pharmacy, Beni-Suef University, Beni-Suef, Egypt

**Keywords:** Intratracheal inhalation, chemotherapy, temozolomide, gold nanoparticles, liposomes

## Abstract

The current study aimed to develop gold nanoparticles (GNPs) and liposome-embedded gold nanoparticles (LGNPs) as drug carriers for temozolomide (TMZ) and investigate the possible therapeutic effects of intratracheal inhalation of nanoformulation of TMZ-loaded gold nanoparticles (TGNPs) and liposome-embedded TGNPs (LTGNPs) against urethane-induced lung cancer in BALB/c mice. Physicochemical characters and zeta potential studies for gold nanoparticles (GNPs) and liposome-embedded gold nanoparticles (LGNPs) were performed. The current study was conducted by inducing lung cancer chemically via repeated exposure to urethane in BALB/C mice. GNPs and LGNPs were exhibited in uniform spherical shape with adequate dispersion stability. GNPs and LGNPs showed no significant changes in comparison to control group with high safety profile, while TGNPs and LTGNPs succeed to improve all biochemical data and histological patterns. GNPs and LGNPs are promising drug carriers and succeeded in the delivery of small and efficient dose of temozolomide in treatment lung cancer. Antitumor activity was pronounced in animal-treated LTGNPs, these effects may be due to synergistic effects resulted from combination of temozolomide and gold nanoparticles and liposomes that may improve the drug distribution and penetration.

## Introduction

Today, nanotechnology is a broad term that offered a new drug delivery system with small objects size between 100–150 nm. Nanotechnology may introduce massive advances in overcoming the problems in drug solubility, stability and improvement of anticancer targeting and distribution (Fanciullino et al., 2013). Nanoparticles consist of different biodegradable materials, such as lipids, liposomes or metals as gold, natural or synthetic polymers and they produced in branched, spherical or shell structures to develop drug delivery system for wide variety of therapeutic agents (Rao & Geckeler, 2011). Gold nanoparticles (GNPs) are metal-based nanoparticles with size ranging between 2 and 100 nm. Many studies confirmed the affinity of either naked or targeted gold nanoparticles, with diameters less than 20, to behave like small molecules. They exhibit a much longer tumor retention time and faster normal tissue clearance, indicating that the well-known enhanced permeability and retention effect (EPR), a unique strength of conventional NPs in tumor targeting, still exists in such small NPs. These advantages enable the AuNPs to target tumor more efficiently without severe accumulation in reticuloendothelial system organs, making them very promising for cancer diagnosis and therapy (Liu et al., 2013). Recently, GNPs are used as nanobiomaterials in molecular imaging and drug delivery systems. GNPs showed high binding affinity with high selective targeting properties to either tissues or cells and passively accumulate at tumor sites due to enhanced permeability and retention (EPR) effects (Vandooren et al., 2016). On the other hand, liposomes are composed of mono or bilayer phospholipid vesicular structures with an aqueous core (Manuja et al., 2012). It is known that liposome is able to change the transdermal behavior of high-molecular-weight drugs in transdermal preparations (Mohammed et al., 2015). Lung cancer is major health problem and leading cause of cancer-related death. Earlier studies reported that the annual increase in lung cancer cases is 82 million beside 1.56 million annual deaths (Torre et al., 2016). Human lung cancer is classified into two major types, that is, nonsmall (NSCLC) and small-cell lung (SCLC) cancer. NSCLC is the most commonly type with 75–85% of lung cancer patients. However, SCLC has been estimated 15–25% of lung cancer patients (Schiller, 2001). Treatment strategies of lung cancer include surgery, radiotherapy and chemotherapy. Selection of the treatment option based on the type, stage and status of the disease within cancerous patient. Chemotherapy has been known as a major component of treatment for all stages of lung cancer (Xu & Le Pechoux, 2015). Cisplatin or carboplatin are the first-line therapy of lung cancer, (Murray & Turrisi 3rd, 2006), while platinum-based therapy is considered as the cornerstone of chemotherapy for the treatment of NSCLC, including the combination between a tubulin binding agent (TBA; paclitaxel, docetaxel) and vinca alkaloids (vinorelbine, vincristine), a camptothecin analog (irinotecan, topotecan), gemcitabine or pemetrexed (MacCallum & Gillenwater, 2006; Pommier et al., 2010). Due to the cancer cell resistance to chemotherapy, management of human neoplasm oppose enormous number of challenges for example adverse effects and therapeutic failure. Consequently, there is a great interest to investigate the effect of wide range of other chemotherapeutic agents such as temozolomide that has been used in the treatment of other cancerous lesions like melanoma. Temozolomide (TMZ) is an alkylating agent, induces a direct damage of DNA through insertion of alkyl groups to both guanine sites at N7, O6 atoms and adenine site at N3 atoms that resulted in unfunctional DNA (Mallick et al., 2015).

The current study aimed to develop gold nanoparticles (GNPs) and liposome-embedded gold nanoparticles (LGNPs) as drug carriers for temozolomide (TMZ) and investigate the possible therapeutic effects of intratracheal inhalation of nanoformulation of TMZ-loaded gold nanoparticles (TGNPs) and liposome-embedded TGNPs (LTGNPs) against urethane-induced lung cancer in BALB/c mice.

## Materials and methods

### Materials

Ethyl carbamate (urethane), gold (ІІІ) chloride and temozolomide (TMZ), L-α-phosphatidylcholine, cholesterol are supplied from Sigma Chemical Company (St Louis, MO). Carcinoembryonic antigen (CEA), insulin growth factor (IGF-1) and cytokeratin 19 fragments (CYFRA21-1) kits were purchased from Alpco Diagnostics (Salem, NH). Interleukin-1β (IL-1β) kit was obtained from Assaypro (St Charles, MO). Tumor necrosis factor-α (TNF-α) was delivered from R&D System Co (Minneapolis, MN). However, alpha-fetoprotein (AFP) was obtained from KAMIYA Biomedical Co (Tukwila, WA). Lactate dehydrogenase (LDH) kit was purchased from Biomed Diagnostic (Cairo, Egypt). Reduced glutathione (GSH) kit was supplied from Sigma-Aldrich (St Louis, MO). Lipid peroxide formation was evaluated as malondialdehyde (MDA) and was purchased from Sigma-Aldrich (St Louis, MO). All other chemicals or solvents were of the analytical or high-performance liquid chromatography (HPLC) grade available.

### Methods

#### Preparation of TMZ-loaded gold nanoparticles (TGNPs)

GNPs were synthesized 2–100 nm by the reduction of an aqueous auric tetrachloride (HAuCl4) solution using sodium citrate as reducing agent (Yonezawa & Kunitake, 1999; Lévy et al., 2004). The nanoparticles size had been controlled by the citrate/gold ratio. The solution was boiled firstly, and then, trisodium citrate (0.04 M) was added until first color changed. The solution was evaluated using UV-visible spectrophotometry and transmission electron microscopy (TEM). The ideal particles were produced upon addition of 25 mL of AuCl_4_ (0.001 M) to trisodium citrate (2.5 mL, 0.04 M). TMZ (6 mg) was dissolved in 1 ml of phosphate buffer solution (pH 7.4), then 500 μL of the formed solution was mixed with 500 μL of gold solution and agitated by ultrasonication for one hour to obtain TGNPs and locally administered by microsprayer in concentration (0.3 mg/100 μL/puff) (Wauthoz et al., 2010).

#### Preparation of liposome-embedded TGNPs (LTGNPs)

According to previous work was carried out by Salem et al. 2015, the optimum ratio of phosphatidylcholine and cholesterol was 1:1 molar ratio (Salem et al., 2015); therefore, phosphatidylcholine (50 mg) and cholesterol (50 mg) were dissolved in amount of chloroform and ethanol mixture (3:2 v/v, 5 mL). Thin film was formed by using rotary evaporator at 50 °C for one hour and to ensure total removal of any traces of solvent; desiccator was used till 2 h (Riaz, 1996). The previously prepared GNPs and TGNPs were added to freshly prepared liposomes at 55 °C, which is above gel–liquid transition temperature will converted to hydrate thin film and agitated for one hour by ultrasonication to obtain LGNPs and LTGNPs, respectively.

#### Physicochemical characterization of particle size and zeta potential of GNPs and LGNPs

Photon correlation spectroscopy (Mastersizer, Malvern, UK) was used for the measurement of mean particle size and polydispersity of GNPs and LGNPs samples. The measurements were carried out at a fixed angle of 90° opposite the incident light source. Small amounts (2 mL) of each sample were added to the quartz cell of the photon correlation spectroscope. Zetasizer 2000 (Malvern Instruments, UK) was used to measure zeta potential of both samples (Broughton-Head et al., 2007).

#### Transmission electron microscope (TEM) imaging

Samples of GNPs, LGNPs, TGNPs and LTGNPs were analyzed using a JEOL JEM-1010 transmission electron microscope (National Research Center, Dokki, Egypt), operating at a voltage of 80 kV. Micrographs were taken at magnifications ranging between 20 000x and 1 200 000x with a Kodak Megaplus digital camera 1.6i (Klimiankou et al., 2005).

### *In vivo* study

Male BALB/c mice (5 weeks, 22–30 g) were purchased from Animal House Colony, Pharmacology and Chemistry Research Center, Misr University and Technology Park, 6th October City, Egypt. Animals were maintained on standard lab diet delivered from Tanta for oils and soups Co. (Tanta City, Gharbia Governorate, Egypt) and housed in filter-top polycarbonate cages in a room free from any source of chemical contamination, artificially illuminated (12-h dark/light cycle) and thermally controlled (25 ± 1 °C) at the Animal House Lab., Faculty of Pharmacy, Beni-Suef university, Bei-Suef, Egypt. All animals received human care and all procedures described below were carried out in accordance with guidelines of ethics committee of faculty of pharmacy, Beni-Suef University, Beni-Suef, Egypt.

#### Induction of lung cancer

Lung cancer was induced by intraperitoneal injection of urethane (ethyl carbamate) (1.5 mg/kg b.w) two times; once at the first and eighth week (Hamzawy et al., 2015).

#### Intratracheal inhalation and drug administration

After anesthesia with intraperitoneal injection of ketamine (12.5 mg/kg) and xylazine (1.5 mg/kg), mice (22–30 g) were intratracheally administered of GNPs, or LGNPs or TGNPs and LTGNP suspension using Microsprayer® IA-1C system (Penn-Century, Philadelphia, PA) (Bivas-Benita et al., 2005).

#### Experimental design

After an acclimatization period of 1 week, animals were treated for 33 weeks and randomly divided into 8 groups (10 mice/group) as follows: group (1): control animals; group (2): animals treated with urethane (1.5 g/kg, i.p) once at the first and eighth week of the treatment period; group (3): animals treated with gold nanoparticles (GNPs 100 μl/puff) by intratracheal inhalation once a week in the latest three consecutive weeks; group (4): animals treated with liposome-embedded gold nanoparticles (LGNPs 100 μl/puff) by intratracheal inhalation once a week in the latest three consecutive weeks; group (5): animals treated with urethane plus GNPs; group (6): animals treated with urethane and LGNPs; group (7): animal treated with urethane and TMZ-loaded gold nanoparticles (TGNPs) (0.3 mg/100 μl/puff) by intratracheal inhalation once a week in the latest three consecutive weeks; group (8): animals treated with urethane and treated with liposomes embedded TMZ-loaded gold nanoparticles (LTGNPs) (0.3 mg/100 μl/puff) once a week in the latest three consecutive weeks.

At the end of treatment period (i.e. day 133), animals were fasted for about 12 h but had free access to water. Blood samples were collected from each animal from the tail vein under ketamine (12.5 mg/kg b.w) and xylazine (1.5 mg/kg b.w) anesthesia (Bivas-Benita et al., 2005). Blood samples were left to clot and the sera were obtained using cooling centrifugation at 3000 rpm for 15 min and freeze at −20 °C until analysis. The sera were used for the determination of carcinoembryonic antigen (CEA), alpha-fetoprotein (AFP), cytokeratin 19 fragments (CYFRA21-1), lactate dehydrogenase (LDH) and insulin-like growth factor (IGF-1) according to the attached manuals of the analytical kits.

After the collection of blood samples, all mice animals were sacrificed by decapitation and their lungs were isolated, weighed, and homogenized in phosphate buffer (pH 7.4) by tissue homogenizer. This homogenate was centrifuged at 1700 rpm at 4 °C for 10 min and the supernatants were stored at −80 °C until analysis of the assessment of lipid peroxide formation (MDA), reduced glutathione (GSH) contents, tumor necrosis factor-α (TNF-α) and interleukin-1beta (IL-1β) levels.

Another portion of lungs were removed and fixed in 10% formalin and embedded in paraffin wax for the histological examination (Drury & Wallington, 1967). Sections were stained with hematoxylin and eosin (H&E), and the stained sections were examined under light microscope according to the method previously described (Bancroft & Gamble, 2008).

### Statistical analysis of data

All data were statistically analyzed by analysis of variance (ANOVA) using the General Linear Model Procedure of the Statistical Analysis System (Stokes et al., 2012). The significance of the differences among treatment groups was determined by Waller–Duncan *k* ratio (Waller & Duncan, 1969). All statements of significance were based on probability of *p* < 0.05.

## Results

### Characterization of GNPs, LGNPs, TGNPs and LTGNP

**Figure 1.**
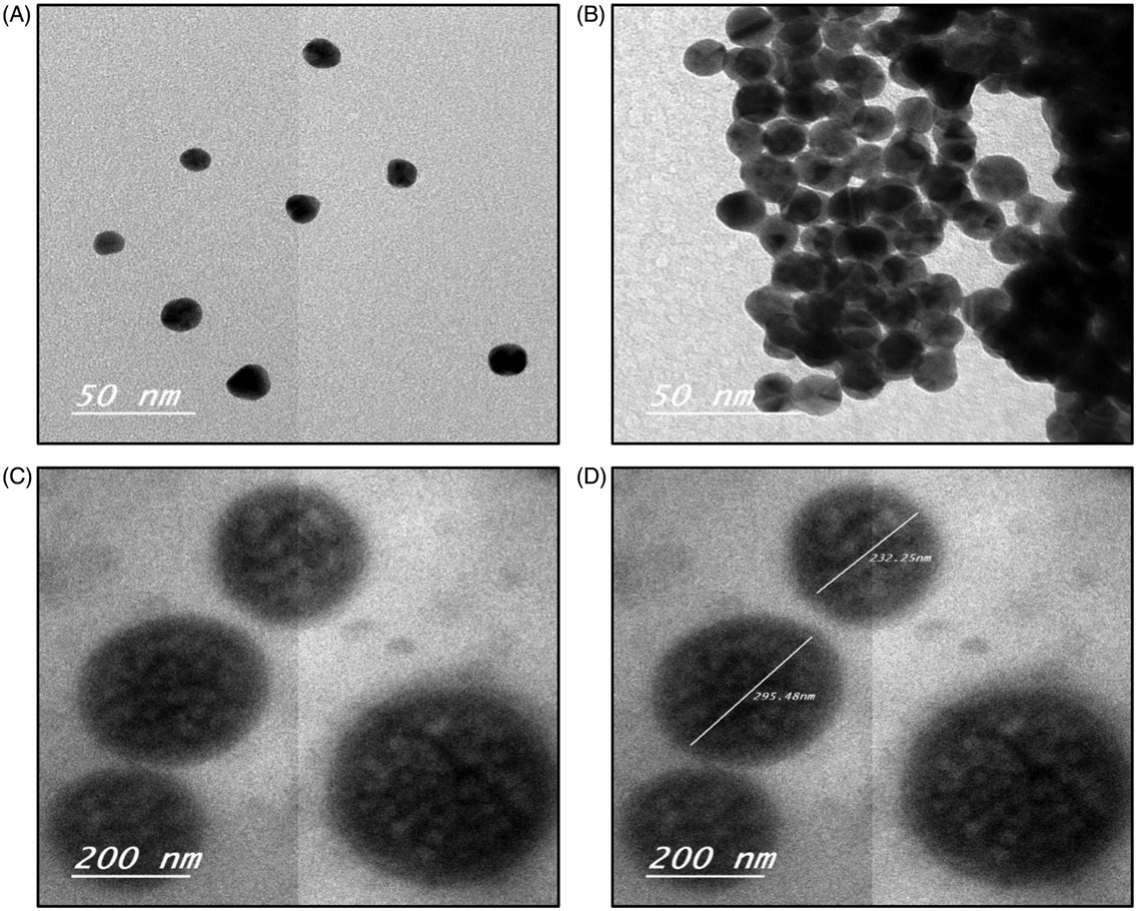
Physicochemical characteristics of the electron microscopic images of blank GNPs and TGNPs (A and B, respectively) (50×); C and D microscopic images of LGNPs and LTGNPs under the electron microscope, respectively (c and d) (200×).

Physicochemical characteristics of the GNPs and LGNPs are depicted in Table 1. Microscopic imaging of the GNPs, LGNPs, TGNPs and LTGNP showed that GNPs and LGNPs possessed spherical shape (Figure 1a and b), while the images of TGNPs and LTGNP exhibited uniform spherical shape more or less like GNPs and LGNPs, respectively (Figure 1c and d). Polydispersity Index (PDI) value of GNPs and LGNPs was (≤1) that indicating narrow size dispersion. Negative zeta potential was observed in value (≤70), indicating adequate dispersion stability of GNPs and LGNPs. Accordingly, GNPs and LGNPs are promising candidate as nanosized vehicle for drug delivery.

**Table 1.** Characterization of blank GNPs and LGNPs.

Group	Zeta potential (mV)	Mean particle size (nm)	PDI
GNPs	−(42.2 ± 5.22)	36.98	0.571
LGNPs	−(69 ± 4.32)	89.04	1.00

### Effect of different treatments on serum LDH activity and tumor markers

Effect of different treatments on serum LDH activity and tumor markers; CEA and AFP are depicted in Table 2. These results indicated that animals treated with urethane showed significant increase in serum LDH activity and CEA and AFP. Animals treated with GNPs or LGNPs alone were comparable to control in serum LDH activity, CEA and AFP. Treatment of GNPs, LGNPs, TGNPs and LTGNPs in animals treated with urethane showed significant improvement in all parameters. These improvements were pronounced in animals treated with LTGNPs more or less like control itself.

**Table 2.** Effect of different treatments on LDH activity and tumor markers in BALB/C mice treated with urethane-induced lung cancer.

Groups	LDH(U/L)	CEA(ng/ml)	AFP(pg/ml)
Control	38.03 ± 3.38	3.36 ± 0.28	4.70 ± 0.44
Urethane	229.43 ± 16.38^a^	74.57 ± 5.16^a^	85.40 ± 5.48^a^
GNPs	65.97 ± 3.27^b^	13.77 ± 0.73^b^	15.83 ± 0.70^b^
LGNPs	53.97 ± 1.79^b^	10.07 ± 0.72^b^	12.53 ± 0.98^b^
Urethane + GNPs	166.00 ± 6.25^a^^,^^b^	44.70 ± 2.98^a^^,^^b^	54.27 ± 3.15^a^^,^^b^
Urethane + LGNPs	123.67 ± 6.22^a^	28.67 ± 1.74^a^	31.17 ± 1.58^a^
Urethane + TGNPs	88.90 ± 3.19^a^^,b^	19.97 ± 0.87^a^^,b^	12.53 ± 0.98^b^
Urethane + LTGNPs	49.53 ± 1.55^a^	6.37 ± 0.48^a^	7.43 ± 0.35^b^

Within each column, means superscript with

^a^ Significantly different from normal control and

^b^ Significantly different from lung cancer control (*p* < 0.05).

### Effect of different treatments on lung homogenate MDA, GSH and inflammatory cytokines

The effect of different treatments on lung homogenate MDA, GSH, TNF-α and IL-1β were depicted in Table 3. Treatment with urethane showed significant increase of MDA, TNF-α and IL-1β and significant reduction of GSH. GNPs and LGNPs treatment showed significant increase in inflammatory cytokines, MDA and reduction of GSH in comparison to control group. Animals treated with GNPs and LGNPs after urethane treatment showed significant reduction of lipid peroxidation (MDA) and inflammatory cytokines; TNF-α and IL-1β and enhancement of GSH in comparison to urethane-treated group. However, treatment with TGNPs and LTGNPs in animals treated with urethane succeeded to improve all parameters and significant improvement was pronounced in animals treated LTGNPs toward to control value.

**Table 3.** Effect of different treatments on lung oxidative stress and inflammatory cytokines in BALB/C mice treated with urethane-induced lung cancer.

Groups	MDA (nmol/g Tissue)	GSH (mg/g Tissue)	TNF-α (Pg/ml)	IL-1β (ng/ml)
Control	138.49 ± 1.34	0.048 ± 0.00072	34.77 ± 3.03	25.33 ± 1.16
Urethane	273.10 ± 1.46^a^	0.019 ± 0.00069^a^	223.67 ± 16.31^a^	209.03 ± 15.5^a^
GNPs	156.66 ± 0.76^a^	0.0440 ± 0.00009^a^	69 ± 2.34^a^	58.87 ± 2.69^a^
LGNPs	154.72 ± 1.66^a^	0.045 ± 0.00012^a^	54.90 ± 2.97^b^	50.13 ± 3.30^b^
Urethane + GNPs	255.32 ± 1.94^a^	0.0215 ± 0.00079^a^	163.63 ± 5.18^a^	151.53 ± 4.94^a^
Urethane + LGNPs	243.49 ± 1.32^a^	0.0029 ± 0.00027^a^	120.33 ± 6.79^a^	108.7 ± 6.81^a^
Urethane + TGNPs	88.90 ± 3.19^a^^,^^b^	0.0359 ± 0.00035^a^	87.80 ± 3.70^a^	77.53 ± 4.02^a^
Urethane + LTGNPs	49.53 ± 1.55^b^	0.0240 ± 0.001^a^^,b^	47.63 ± 1.88^b^	38 ± 1.70^b^

Within each column, means superscript with

^a^ Significantly different from normal control and

^b^ Significantly different from lung cancer control (*p* < 0.05).

### Effect of different treatments on serum CYFRA 21-1 and IGF-1 level

**Figure 2.**
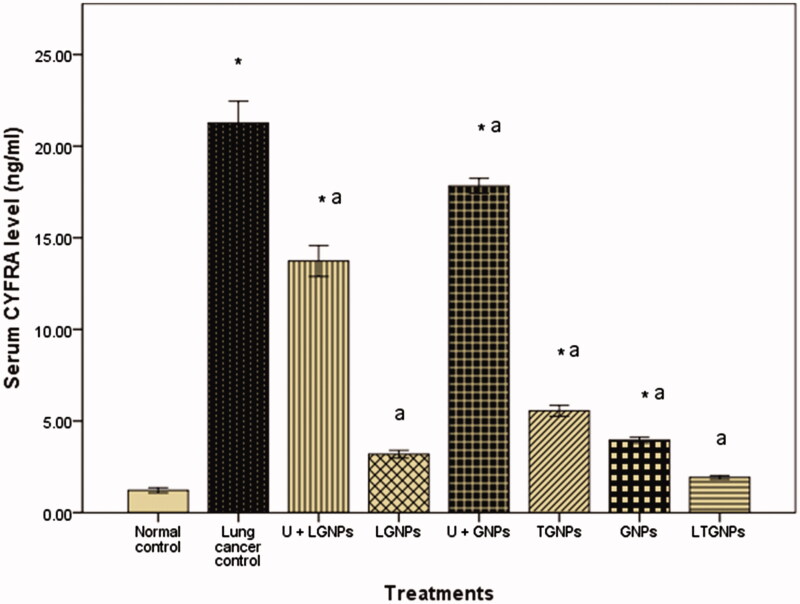
Effect of TMZ nanoparticles on serum cytokeratin 19 fragments (CYFRA 21-1) level in BALB/C mice treated with urethane-induced lung cancer. Within each bar, means superscript with * significantly different from normal control and ^a^significantly different from lung cancer control (*p* < 0.05).

**Figure 3.**
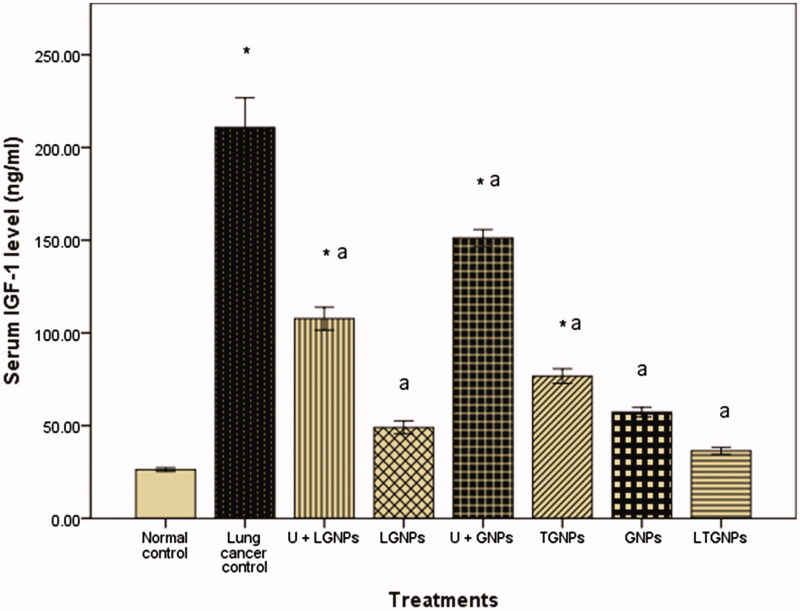
Effect of TMZ nanoparticles on serum insulin-like growth factor-1 (IGF-1) level in BALB/C mice treated with urethane-induced lung cancer. Within each bar, means superscript with * significantly different from normal control and ^a^significantly different from lung cancer control (*p* < 0.05).

The existing results of the current study proved that urethane induced significant increase in serum of CYFRA 21-1 and IGF-1. Treatment with GNPs and LGNPs alone showed no significant difference in comparison to control group. Mice treated with urethane then treated with GNPs and LGNPs revealed significant reduction in serum of CYFRA 21-1 and IGF-1, but still higher than untreated control group. On the other hand, treatment with TGNPs and LTGNPs in animals pretreated with urethane showed significant reduction in the serum of CYFRA 21-1 and IGF-1 more or less like control. LTGNPs succeeded to improve the serum level of CYFRA 21-1 and IGF-1 toward control value (Figures 2 and 3).

### Histological examinations

**Figure 4.**
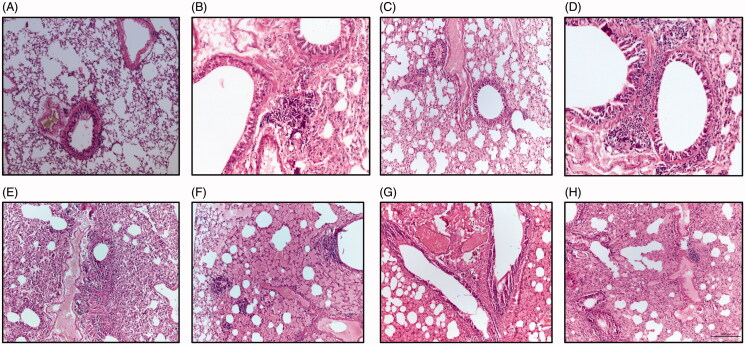
A photomicrograph of lung sections of control group (A) showing normal epithelization of bronchi and bronchioles with normal alveoli and normal configuration of thymus; (B) urethane-treated mice exhibiting foccal aggregation of neoplastic epithelial cells around the bronchioles and marked pleomorphic and advanced mitotic activities; (C) GNPs-treated mice showing few peribronchial proliferations of fibroblast cells and hemolysis of blood vessels; (D) Mice treated with LGNPs showing few peribronchial proliferations of fibroblast cells together with few lymphocytes and macrophages and hemolysis of blood vessels; (E) treated with Urethane + GNPs demonstrating few peribronchial and perivascular aggregation of neoplastic cells and severe proliferation of pneumocytes and occlusion of air alveoli and slightly hemolysis of blood vessels; (F) lung sections from mice treated with urethane + LGNPs exhibiting group of parabronchial aggregation of neoplastic cells with marked pleomorphic and advanced mitotic activities and notice congestion and edema in the alveoli; (G) lung section from mice animals treated with Urethane + TGNPs showing hyperplasia of bronchial epithelial cells and severe hemolysis of blood vessels with mild proliferation of pneumocytes among alveoli; (H) lung section from animal treated with urethane + LTGNPs display paravascular aggregation of lymphocytes and few fibroblast cells and hyperplasia of bronchial epithelial cells and marked alveolar edema with hemolysis in blood vessels. (H&E X 200).

The biochemical results obtained in the current study were confirmed by histopathological examinations of lung tissues. Microscopic examinations of lung section of control mice showed normal histological structure that indicated by normal epithelization of bronchi, bronchioles and alveoli without incidence for tumor nodules or microscopical lesions (Figure 4A). Photomicrograph of lung section of animals treated with urethane exhibited focal aggregation of neoplastic epithelial cells around the bronchioles and marked pleomorphic and advanced mitotic activities (Figure 4B). The lung section from mice treated with GNPs only illustrated few peribronchial proliferations of fibroblast cells and hemolysis of blood vessels (Figure 4C). The lung section of lung from animals treated with LGNPs only exhibited few peribronchial proliferations of fibroblast cells together with few lymphocytes, macrophages and hemolysis of blood vessels (Figure 4D). Microscopic examination of lung section from animals treated with GNPs after urethane treatment showed few peribronchial and perivascular aggregation of neoplastic cells and severe proliferation of pneumocytes (cells among alveoli) and so occlusion of air alveoli associated with slightly hemolysis of blood vessels (Figure 4E). The lung section from mice treated with urethane then LGNPs showed dense of parabronchial aggregation of neoplastic cells with marked pleomorphic, advanced mitotic activities beside congestion and edema around the alveoli (Figure 4F). A photomicrograph of lung section from mice treated with TGNPs after urethane treatment revealed hyperplasia of bronchial epithelial cells associated with severe hemolysis of blood vessels beside hemosidrosis and mild proliferation of pneumocytes among alveoli (Figure 4G). The lung section of mice treated with urethane and LTGNPs showed paravascular aggregation of lymphocytes, few fibroblast cells accompanied with hyperplasia of bronchial epithelial cells with marked alveolar edema and hemolysis in blood vessels (Figure 4H).

## Discussion

Nanomedicine is designing and development materials, used as vehicle or carrier in drug delivery systems or diagnostic tools and measured in nanoscopic scale (Chen et al., 2016). Nanobased medicine has witnessed rapid development in the last decade in diagnosis, imaging, detection and treatment of various types of cancer (Nie, 2010). Nanoparticles showed optimal physicochemical properties to improve both pharmacokinetic, pharmacodynamics profiles of the drugs with significant enhancement of therapeutic properties (Wu et al., 2011). Loading of drugs in nanoformulation alter their biodistribution characters of the parent drugs such as increase the drug stability; enhance the drug stand on the blood circulation and effect of permeability and retention (EPR) in cancer site. On the other hand, nano-based medicine in treatment of lung cancer undergo various challenges as specific size of the distribution, ability to retain for sufficient time at targeted site and clearance (Hussain, 2016). Wide ranges of nanomaterials have been used as nanocarriers for example organic, inorganic, lipid, protein or glycan compounds as well as synthetic polymers in development of new cancer chemotherapy (Prabhakar et al., 2013). In the current study, gold nanoparticles and liposomes were constructed as carriers for temozolomide (TMZ) to investigate the potential therapeutic effect of intratracheal inhalation of TGNPs and LTGNPs against urethane-induced lung cancer in BALB/C mice.

The result of the current work showed that GNPs and LGNPs possess spherical shape with small particle size (≤89 nm) that indicated these systems are useful tool as delivery system for chemotherapeutic drugs with ideal tumor accumulation properties (Park et al., 2006; Iodice et al., 2016) and improve the possibility of penetration into the cells in comparison to other formulations (Chithrani et al., 2006). The selected dose of urethane was based on previous work was conducted by (Hamzawy et al., 2015). The results of the current study revealed that urethane induced significant increase of LDH activities that indicated by increase in cellular proliferation and cancer promotion.(Liu et al., 2015b) It has been well documented that LDH plays an essential role in development, invasion and metastasis of lung cancer via accumulation of F-fluorodeoxyglucose (F-FDG) through increase in the expression of glucose transporters type 1 (GLUT1) (Zhou et al., 2014). LDH is prognostic marker for distinguishing among the different stages of lung cancer and other solid tumor with whole body extent (Lee et al., 2015). On the other hand, significant increase in tumor markers, CEA and AFP was in agreement with previous studies. The increase in serum of AFP may be due to formation of active metabolite; vinyl carbamate, which is potent mutagen via interaction with DNA to form 2-oxoethyl adducts as vinyl chloride (Willder et al., 2012). AFP is specific biomarker of hepatocellular carcinoma and synthesis in the fetal stage of germ cells. Earlier study reported that active metabolite; vinyl carbamate-induced hepatic lesions and metastasis (Surh et al., 1998). Patients with primary lung carcinoma and testicular metastasis showed significant elevation of AFP (Yamagata et al., 2004). Urethane showed significant increase in CEA due to promotion of cellular proliferation and pulmonary neoplasia (Cekanova et al., 2009). The results of the current study revealed that urethane increase serum Insulin-like growth factor 1 (IGF-1) through up-regulation of NF-кB and downregulation of other tumor suppressor genes (Pandey & Gupta, 2012; Treda et al., 2013). Earlier studies postulate that IGF-1-induced lung adenocarcinoma through activation of MAPK- and AKT-signaling pathways (Tang et al., 2013). Data of the present work demonstrated that urethane is a potent carcinogen and induce CYFRA21-1 level, which is the specific epithelial cell markers and very sensitive for non-small cell lung cancer patients with poor prognosis (Wieskopf et al., 1995; Takahashi et al., 2010). Urethane-induced alteration of lipid peroxidation and oxidative stress that indicated by significant increase in MDA level and reduction of GSH activity. This could be attributed to increase the intrinsic ROS generation via release of electrophilic species with permanent dysfunction of mitochondria at lung cells (Kowaltowski et al., 2009). Mitochondrial dysfunction and oxidative stress of repeated exposure to urethane associated with over stimulation of IFN-γ-producing cells, CD11b + Gr-1 + phenotype (Pandey & Gupta, 2012), overexpression of NF-кB, COX-2, STAT3, IL-6 and cyclin D1 resulted in chronic inflammation and lung cancer (Narayan & Kumar, 2012). The data of the present work showed that urethane treatment resulted in significant increase of inflammatory cytokines; TNF-α, IL-1β in comparison to control group. The results were in agreement with earlier studies that postulated urethane induce over expression of key molecules such as NF-κB, Stat3, pStat3; IL-1β, those play a major role in chronic inflammation and lung adenocarcinoma (Bernert et al., 2003). The molecular mechanism of urethane-induced lung cancer based on integration of TNF signals with NF-кB that accelerate tumor progression (Nakahara et al., 2013). Intratracheal inhalation of either GNPs or LGNPs showed non-significant difference in LDH, CEA and AFP in comparison to control group. These results of the present work were in agreement with previous findings that indicated safety of gold nanoparticles or liposomes as a drug carrier (Correard et al., 2014; Qian et al., 2015). On the other hand, GNPs or LGNPs treatment induced significant changes of MDA, GSH. The results of the current study were in agreement with the earlier report that indicates gold nanoparticles may induce the oxidative damage with alteration of oxidative parameters and energy metabolism (Ferreira et al., 2015). In the same line, animals treated with GNP or LGNPs alone exhibit normal CYFRA21-1 and IGF-1 more or less like control. These results indicated ability of gold nanoparticles to inhibit cell proliferation and cell cycle in G1-phase via upregulation of cell-cycle cyclin-dependent kinase inhibitor proteins p21 and p27 (Bhattacharya et al., 2007).

In the current study, treatment with GNP or LGNPs plus urethane showed significant improvement in all tested biochemical parameters. GNPs induced apoptosis and necrosis in lung cancer cells due to its cytotoxic effect via increase of intracellular reactive oxygen species (ROS) and regulation of cellular glutathione in lung cancer cells by conjugation with glutamate cysteine ligase catalytic (GCLC-specific siRNA) (Liu et al., 2015a). The improvement was pronounced in animals treated with LGNPs later than urethane treatment that may be attributed to high accuracy in dose versus time-release of gold nanoparticles (Zhang et al., 2016). The results of the current study showed that treatment of TGNPs plus urethane exhibited significant improvement of all biochemical parameters more or less like control. These results were in agreement with preceding study that indicated loading temozolomide into GNPs resulted in synergistic effects in regarding to apoptotic effects beside reversing acquired resistance to chemotherapy (Orza et al., 2013). Loading of TMZ into gold nanoparticles embedded into liposomes (LTGNPs) showed improvement in all measured parameters to normal value of control group. These results were similar to previous study that reported liposomes enhance the therapeutic effect, prolong *in vivo* circulation and increase the bioavailability and AUC to the incorporated drugs (Gao et al., 2015). The outcomes of the present work revealed that treatment of LTGNPs showed advanced restoration toward normal values. Liposomal formulation improve drug stability and increase drug dispersion of TMZ (Kojima et al., 2008). Previous studies reported that liposomal formulations improve a drug’s pharmacokinetics and biodistribution, prolonged drug circulations, enhanced permeability and retention (EPR) effect of anticancer drugs (Guo & Huang, 2014). On the other hand, the localization treatment via intratracheal administration enhance the drug efficacy and reduced the systemic effect in comparison to other forms of drug delivery (Xie et al., 2010). The biochemical results were confirmed with histological examinations of lung tissues that typical reported in the previous literatures (Stathopoulos et al., 2007; Li et al., 2015).

## Conclusion

The present study focused on designing drug carrier capable of delivering small and effective dose of temozolomide (TMZ) via intratracheal inhalation by loading into either GNPs or liposome-embedded gold nanoparticles (LGNPs). GNPs and LGNPs are promising candidates as drug carrier for temozolomide (TMZ). Intratracheal inhalation of TGNPs and LTGNPs showed significant improvement of drug efficacy associated with reduction of systemic toxicity. LTGNPs succeeded to improve all measured parameters with advanced restoration to normal values. The results of the *in vivo* study indicated that LTGNP provide synergistic effect through combination of gold nanoparticles and TMZ beside the enhancement of drug permeability and retention (EPR) effect.
